# Effects of Culture on Musical Pitch Perception

**DOI:** 10.1371/journal.pone.0033424

**Published:** 2012-04-11

**Authors:** Patrick C. M. Wong, Valter Ciocca, Alice H. D. Chan, Louisa Y. Y. Ha, Li-Hai Tan, Isabelle Peretz

**Affiliations:** 1 The Roxelyn and Richard Pepper Department of Communication Sciences & Disorders, and the Knowles Hearing Center, Northwestern University, Evanston, Illinois, United States of America; 2 Department of Otolaryngology—Head & Neck Surgery, Northwestern University, Chicago, Illinois, United States of America; 3 School of Audiology and Speech Sciences, University of British Columbia, Vancouver, British Columbia, Canada; 4 Department of Linguistics and Multilingual Studies, Nanyang Technological University, Singapore, Singapore; 5 School of Humanities, University of Hong Kong, Hong Kong, China; 6 International Laboratory for Brain, Music and Sound Research (BRAMS), Université de Montréal, Montreal, Quebec, Canada; Max-Planck Institute of Neurobiology, Germany

## Abstract

The strong association between music and speech has been supported by recent research focusing on musicians' superior abilities in second language learning and neural encoding of foreign speech sounds. However, evidence for a double association—the influence of linguistic background on music pitch processing and disorders—remains elusive. Because languages differ in their usage of elements (e.g., pitch) that are also essential for music, a unique opportunity for examining such language-to-music associations comes from a cross-cultural (linguistic) comparison of congenital amusia, a neurogenetic disorder affecting the music (pitch and rhythm) processing of about 5% of the Western population. In the present study, two populations (Hong Kong and Canada) were compared. One spoke a tone language in which differences in voice pitch correspond to differences in word meaning (in Hong Kong Cantonese, /si/ means ‘teacher’ and ‘to try’ when spoken in a high and mid pitch pattern, respectively). Using the On-line Identification Test of Congenital Amusia, we found Cantonese speakers as a group tend to show enhanced pitch perception ability compared to speakers of Canadian French and English (non-tone languages). This enhanced ability occurs in the absence of differences in rhythmic perception and persists even after relevant factors such as musical background and age were controlled. Following a common definition of amusia (5% of the population), we found Hong Kong pitch amusics also show enhanced pitch abilities relative to their Canadian counterparts. These findings not only provide critical evidence for a double association of music and speech, but also argue for the reconceptualization of communicative disorders within a cultural framework. Along with recent studies documenting cultural differences in visual perception, our auditory evidence challenges the common assumption of universality of basic mental processes and speaks to the domain generality of culture-to-perception influences.

## Introduction

The present study examines how differences in cultural backgrounds affect the way in which people perceive auditory signals. Specifically, we focus on language, a prominent aspect of culture, and on pitch, a perceptual attribute that not only forms the basic building blocks of music, but also conveys crucial information about talker identity, spoken emotion, and in some instances word meaning (in the case of tone languages). We investigate how speakers of a language in which pitch is used to mark word meaning (Cantonese Chinese speakers) differ from those who do not speak such a language (English and French speakers) when processing musical pitch.

Definitions of culture often include systems of communication (e.g., language), visual and performing arts (e.g., music), religions, and social norms [1]. Often excluded from these definitions are basic mental processes, such as sensory perception, memory and attention [2], [3]. Interestingly, there is now growing evidence suggesting that some aspects of perceptual processing also differ among people from different societies [4]–[6]. Much of the evidence comes from studies of visual recognition memory and eye-tracking, in which participants view pictures with a clear focal object in the context of a background scene [7]. Westerners (mostly comprised of North Americans, but also including Western Europeans) in these studies have a higher rate of attending to the foreground object whereas East Asians (Koreans, Japanese, and Chinese) attend to both the foreground object and the background. In addition, cultural differences in worldview, representations of self, and even thinking styles have been documented extensively: East Asians tend to be more collective, interdependent, and holistic, while Westerners tend to be more individualistic, independent, and analytic [8]–[10]. These broader and higher-level cultural differences have been used to explain differences in visual perception. For example, because East Asians view the world more holistically, they see both the background and foreground object in a picture, and because Westerners are more analytic, they focus more on the salient foreground object alone [11].

The present study focuses specifically on one prominent aspect of culture, language, and examines how experience in speaking different languages may affect auditory perception, which is itself the foundation of spoken language processing. We examine speakers of tone and non-tone languages. In tone languages, pitch is used to signal word meaning in addition to phrasal meaning through intonation (“speech melody”), whereas in non-tone languages such as English and French, pitch is used to signal intonation only. Languages that use lexical pitch are estimated to account for about 70% of the world's languages [12], and include Cantonese and Mandarin Chinese. For example, in Mandarin, the syllable /ma/ can mean ‘mother’ or ‘to scold’ depending on whether it is spoken with a high or falling pitch pattern, respectively. In languages where pitch is used at the phrasal level, changing pitch cannot change word meaning, but can signify the speaker's emotion and intent, as well as the declarative/interrogative status of the phrase. Participants in our study included Cantonese speakers recruited in Hong Kong, China and Singapore, as well as English and French speakers recruited in Montreal, Canada and Singapore. We focused on a dominant aspect of auditory processing, pitch, with rhythm as a control condition, by using the On-Line Identification Test of Congenital Amusia [13]. Previous research has been conducted on amusia, a condition affecting music processing which is sometimes known as tone deafness [14] and dysmelodia [15]. In typically developing children, sensitivity to aspects of music including pitch and rhythm can be observed before one year of age [16]; however, congenital amusia is found in about 4–6% of the Western (non-tone language speaking) population [13], [15], and is associated with neural [17]–[19] and genetic [20], [21] factors.

The ascending auditory pathway contains neural structures that are associated with increasing levels of complexity of sound processing [22]. What makes the connection between musical processing (including amusia) and lexical tone processing a particularly interesting line of inquiry is that it offers a unique opportunity for understanding the convergence, divergence, and interactions of types of auditory processing along this pathway, especially in relation to the association between language and music [23], [24], [25], [26], [27], [28], [29]. More specifically, as pitch is used as a primary functional unit in both music and tone languages, questions arise as to whether extensive experience with one type of pitch processing may influence another type of pitch processing, and under what circumstance (or at which level of the auditory pathway) mutual influence occurs. This question has gained much attention in recent years, including studies that found musical training to facilitate lexical tone perception [30], tone language learning [31], [32], [33], and the encoding of lexical tone patterns in the rostral brainstem [24], [34], [35], [36]. What remains to be investigated is whether extensive experience with tone languages influences musical pitch processing, including on listeners with no obvious pitch deficits. Acknowledging the fact that “music” has an intricate combinatorial property that spans beyond its basic building block of pitch, we define musical pitch processing as the processing of musical melodies that require the integration of local and longer distance pitch syntactic information. This definition also acknowledges the important contributions of pitch intervals [37], [38], [39], [40] and rapid frequency modulations [41], [34], [35], [36] that when combined following musical combinatorial rules, build music. We believe an investigation of language-to-music relationship investigations will provide evidence for a true double association of pitch processing in music and speech.

**Figure 1:**
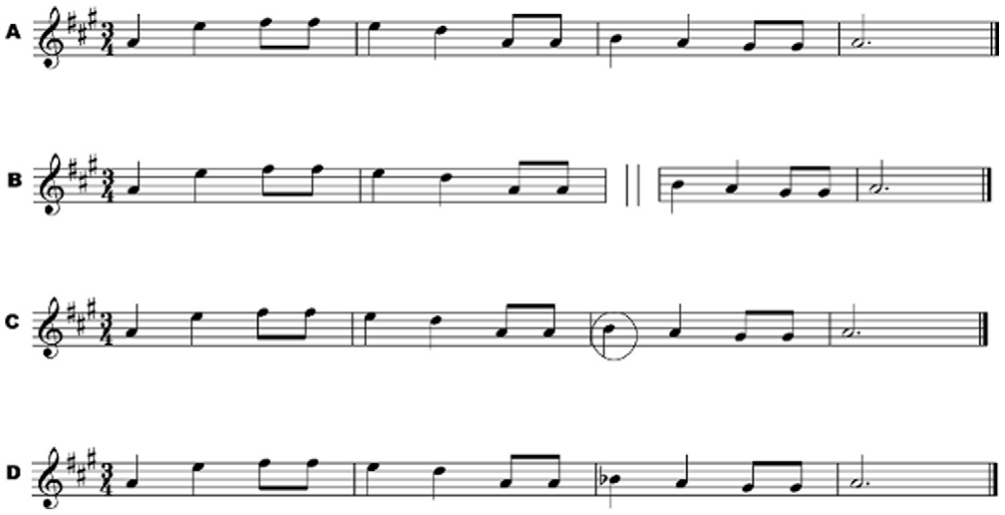
Example of a melody in the On-Line Identification Test of Congenital Amusia. Conditions for the melody are shown: no incongruity (A), time incongruity (B: ∥ refers to the silence of 5/7 of the beat duration), mistuned pitch incongruity (C: the circled note refers to the mistuned pitch), and out-of-key pitch incongruity (D).

Participants in the current study completed the On-Line Identification Test of Congenital Amusia (Hong Kong version) in Experiment 1, the original version of which was normed in Montreal on native Canadian English and Canadian French speakers. This test has three conditions [13] that require detection of incongruities in short melodies composed of Western tonal keys and rhythmic structures. The Off-beat condition assesses rhythm perception and requires participants to detect melodies in which temporal incongruities are present. The Out-of-Key and Mistuned conditions both assess pitch perception, with the former containing notes that are tuned correctly but violate the tonal (syntactic) relationships given the key of the melody, and the latter containing notes that are mistuned by a quarter tone in addition to syntactic violations. The psychometrics of this test have been reported elsewhere [13]. Sample melodies from each condition are presented in Figure 1. Participants in Hong Kong completed a version of this test in which the written instructions were translated into Chinese, and their performance was compared with those of the Montreal participants. Given that pitch is used more extensively in tone than in non-tone languages, we investigate not only if amusia exists in tone language speaking populations [42], [43], but also whether tone language speakers have better pitch (but not rhythm) perception skills relative to non-tone language speakers as a group regardless of whether they demonstrate poor music perception abilities. We found that Cantonese speakers show enhanced ability in processing musical pitch in the Out-of-Key condition relative to those who speak a non-tone language. As will be discussed, both groups of participants performed at near-ceiling in the Mistuned condition in Experiment 1. As such, we performed a follow-up experiment (Experiment 2) by increasing the difficulty of the task to determine whether group differences exist in judging mistuned musical notes.

## Experiment 1

### Methods

#### Ethics Statement

All experimental procedures for both experiments were approved and all participants provided informed consent in accordance with the Institutional Review Boards of Northwestern University, University of Hong Kong, and Nanyang Technological University of Singapore.

#### Participants

Hong Kong participants were recruited by posting an advertisement on the University of Hong Kong internet communication system during spring-summer 2007 and winter-spring 2008. Potential participants were asked to encourage their friends and family members outside of the university to participate. All Hong Kong participants self-reported that Chinese was their native language and that they had no known hearing or brain deficits. Characteristics of the Canadian participants were reported in Peretz et al. (2008) [13]. Comparisons of basic characteristics of the Hong Kong and Canadian participants are described in the Supporting Information S1.

Because normative data of the Hong Kong version of the On-Line Identification Test of Amusia have not been reported previously, we include such data in the Supporting Information S1. Table S1 in the Supporting Information S1 summarizes the characteristics of the Hong Kong participants. In total, 446 participants completed the entirety of the test, with 408 of them being younger than 40 years old. Because younger and older adults were consistently found to differ in their performance [13], [44], and because very few Hong Kong participants were 40 years old and older, direct comparisons of the Hong Kong and Canadian participants only included those who were younger than 40 years old. However, the Supporting Information S1 provides details of the performance from all Hong Kong participants (Figure S1 & Table S2).

#### Methods and Materials

The test materials consisted of a translated version of the On-Line Identification of Congenital Amusia reported in Peretz et al. (2008) [13]. A native Cantonese speaker from Hong Kong who is fluent in both Cantonese and English translated the test from English to idiomatically appropriate Chinese (traditional Chinese characters are used per conventions in Hong Kong). Eight native Cantonese speakers from Hong Kong proofread the translation and ensured cultural appropriateness for Hong Kong. After several revisions, the final version of the Online Identification of Congenital Amusia (Hong Kong Version) was used for testing. The test procedures are identical to Peretz et al. (2008) [13] and will only be described briefly here. Participants completed the test at a location convenient to them over the internet using a standard web browser. They made (in)congruity judgments by using a computer mouse to indicate ‘yes’ or ‘no’ in three test conditions: Off-beat, Out-of-Key, and Mistuned. In the Off-beat condition, half the melodies contained a silence of 5/7 of the beat duration prior to the first downbeat in the third bar of the four-bar melody; in the Out-of-Key condition, the pitch of the same downbeat violated the tonal/syntactic relationship when considering the rest of the melody; and in the Mistuned condition, the same pitches were mistuned by a quarter tone with the addition of a tonal/syntactic violation. In other words, melodies in the Mistuned condition are incongruent in two ways. Figure 1 illustrates the three conditions (taken from Figure 1 of Peretz et al., 2008) [13]. Participants were first tested with the “off-beat” condition followed by the “mistuned” condition and finally the “out-of-key” condition. In each condition, participants were presented with 24 melodies (12 containing no incongruity and 12 containing an incongruity) one at a time, in a random but fixed order. The task was to detect whether an incongruity occurred in each melody, by way of clicking a “yes” button whenever there was an anomaly, and a “no” button when there was none. Participants received 2 examples before each condition and were provided with feedback after these two trials only. The entire test lasted about 15 minutes. After the test, participants were asked to answer a series of questions concerning their health, musical, and educational history.

#### Calculation of Adjusted Scores

When comparing amusic participants from the two groups, we adjusted their Out-of-Key scores by degrees of musical training based on the general linear model adjusted for the covariate. The specific formula includes:

ŷ = a+B * (degrees of musical training), where ŷ is the fitted value, a is the intercept, and B is the linear coefficient for musical training.

Adjusted Score = Raw Out-of-Key Score−ŷ

### Results

We report here results from participants between 18 and 40 years old, which include 408 participants from Hong Kong (267 females) and 154 participants from Canada (99 females). Table 1 summarizes the results from the two groups of participants. The Supporting Information S1 provides a detailed report. We first report comparisons of the two participant populations broadly, with a focus on the amusic individuals in the second section.

**Table 1:** Comparison of Hong Kong and Canadian Younger Participants (18–39 years old only).

	Hong Kong	Canada
	*N* = 408	*N* = 154
Male/female (% female)	141/267 (65.4)	55/99 (64.3)
Mean age (range)	23.5 (18–39)	24.6 (18–39)
Mean education year (range)	16.0 (7–33)	17.0 (11–30)
Musical training level* (range)	4.1 (1–5)	3.1 (1–5)
Off-beat (SD)	84.0% (8.8)	84.2% (8.7)
Mistuned (SD)	94.2% (6.6)	94.2% (7.4)
Out-of-key (SD)	91.1% (8.5)	86.3% (11.3)
Global score (SD)	89.8% (5.7)	88.2% (7.1)
2 SD Cut-off	78.4%	73.9%
% Amusic	3.9% (16/408)	5.2% (8/154)

The 2 standard deviations (SD) cutoff for amusia is based on the Global Score.

* Musical training is classified into 5 levels: 1 = less than one year, 2 = 1–3 years, 3 = 4–6 years, 4 = 7–10 years, and 5 = more than 10 years.

#### General Populations

**Figure 2:**
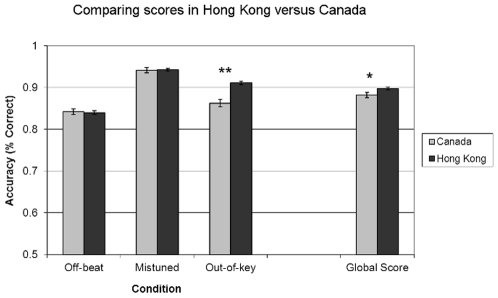
Comparisons of Hong Kong and Canadian participants (younger participants only) on the Online Amusia Test. Error bars indicate standard error of the mean. Only the Out-of-Key condition shows a significant group difference (**p<.001); note that after controlling for age, education, and musical training differences, the group difference in the Out-of-Key condition remained. The Global Score also showed a significant group difference (*p = .007).

Figure 2 shows group performance for each condition and the Global Score (the Global Score is the composite of the three conditions). To assess group differences, a 2×3 mixed-effects ANOVA was conducted and revealed a main effect of condition [F (2, 1120) = 234.392, p<.001, η*_p_* ^2^ = .295], a main effect of group [F (1, 560) = 7.389, p = .007, η*_p_* ^2^ = .013], and a significant group×condition interaction [F (2, 1120) = 18. 650, p<.001, η*_p_* ^2^ = .032]. Posthoc One-Way ANOVAs performed on each condition revealed no significant group difference in the Off-beat [F (1, 560) = .073, p = .788] and Mistuned [F (1, 560) = .017, p = .896] conditions, but a significant difference in the Out-of-Key condition F (1, 560) = 30.261, p<.001, η*_p_* ^2^ = .051] (significant after Bonferroni correction). Thus, the main effect of group was driven by the substantial difference in the Out-of-Key condition. A one-way ANOVA also revealed a significant group difference on the Global Score [F (1, 560) = 7.389, p = .007, η*_p_* ^2^ = .013]. It is worth pointing out that over 82% of the participants in each group correctly classified 22 out of 24 stimuli in the Mistuned condition, which suggests that the lack of group difference was likely due to a ceiling effect.

**Figure 3:**
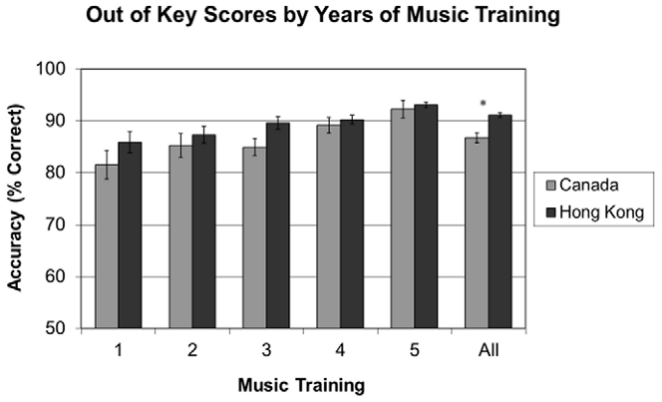
Hong Kong and Canadian younger participants' Out-of-Key performance divided by group. Musical training is classified into 5 levels: 1 = less than one year, 2 = 1–3 years, 3 = 4–6 years, 4 = 7–10 years, and 5 = more than 10 years.

The two groups of participants also differed in age, education, and level of musical training (see Supporting Information S1 for details). Therefore, it is important to ascertain that the group difference in the Out-of-Key condition still remained after these factors were controlled. We conducted an ANCOVA with the Out-of-Key score as the dependent variable, group as a random variable, and age, education, and musical training as covariates. The main effect of group remained [F (1, 540) = 5.913, p = .015, η*_p_* ^2^ = .011]. Figure 3, which shows participants' Out-of-Key performance divided by musical training, demonstrates that both groups were affected by musical training, but overall Hong Kong participants outperformed Canadian participants. This shows that speakers of a tone language have increased ability in musical pitch (but not rhythm) processing.

It is worth noting that some of our data did not show a normal distribution (see Supporting Information S1). Although the use of parametric statistics was still justified because of our large sample size [45], we performed a non-parametric test (Independent-Samples Mann-Whitney U Test) on all measures, including the Global Score, to further validate our results. Our initial findings were replicated. There was no statistical group difference in the Off-Beat (p = .962) and Mistuned (p = .601) conditions, but a significant group difference was found in the Out-of-Key condition (p<.0001) and the overall Global Score (p = .016).

#### Amusic Participants

In defining amusia, it is a common practice to consider participants' overall performance in both pitch and rhythm processing [13], [44]. Following the same procedures as previous research [13], participants' performance in all three conditions were averaged to form a Global Score and amusia was defined as performance below two standard deviations of the mean of this Global Score. Using this criterion, Hong Kong and Canadian participants with a Global Score below 78.4% and 73.9%, respectively, were classified as amusic. We found 3.9% of the Hong Kong participants to be amusic, compared to 5.2% of the Canadians. Note that the use of 2 standard deviations as a criterion simply reflects our effort to conform to previous studies. Alternatively, we also employed a criterion-based approach with a global score of 70%, 80%, and 90% (selected arbitrarily) and found a higher percentage of the Canadian participants to perform below all of these cutoffs (Table 2).

**Table 2:** Percentage of participants from each group who performed below three arbitrary cutoff criteria (based on Global Score).

	70%	80%	90%
Hong Kong	1.0%	3.9%	34.3%
Canada	2.6%	7.8%	44.2%

As the distributions of participant populations were not normal in our study nor in previous studies (see Supporting Information S1), a standard deviation does not readily translate into a fixed percentage of the populations for a meaningful quantitative comparison of amusic participants. This fact is complicated by group differences in degree of musical training. In order to understand and compare differences of the amusic participants in the two groups, we adopted two statistical procedures. We first derived a set of scores adjusted for differences in degrees of musical training (see Methods) from the two groups. We only focused our analyses on the Out-of-Key condition as it is the only condition that showed group differences as discussed. We then compared the performance of the bottom 5% of the participants based on the adjusted scores derived from the Hong Kong (n = 20) and Canadian (n = 7) group and found the lowest-performing (amusic) Hong Kong participants to significantly outperform the Canadian participants [One-way ANOVA: F(1, 25) = 8.448, p<.008, η*_p_* ^2^ = . 253].

#### Responses to Self-Assessment Questionnaire

Peretz et al. (2003; 2008) [13], [31] observed that three questions on their questionnaire were particularly important for identifying amusic participants. These descriptions are: “I cannot recognize tunes without the help of the lyrics,” “I cannot tell if I sing out of tune” and “I have been told I sing out of tune.” Of the three, the participant's ability to detect whether someone else is singing out-of-tune was reported to most effectively distinguish amusic from non-amusic individuals. Table 3 summarizes the results and shows that in general, more amusics than non-amusics from both populations self-reported ‘yes’ to all three questions. However, this pattern is much less pronounced in the Hong Kong group.

**Table 3:** Percentage of responses (proportion of participants) to questions relevant for the identification of amusic individuals.

	Hong KongAmusic	Hong KongNon-Amusic	CanadaAmusic	CanadaNon-Amusic
Unable to detect when someone sings out-of-tune	25%	4%	85%	0%
Can rarely recognize a very familiar melody without the help of lyrics	13%	2%	74%	8%
Sings out-of-tune	75%	30%	93%	43%

Amusia is defined by 2 SD below the mean of Global Scores normed on each population. Data from the Canadian participants were taken from Peretz et al. (2008) [13].

### Discussion

We found evidence in Experiment 1 that Hong Kong participants outperformed Canadian participants in the Out-of-Key condition but not in the Off-Beat condition, suggesting that Hong Kong participants have elevated melodic pitch perception abilities. Both groups of participants performed at near-ceiling in the Mistuned condition. To determine whether group differences exist in judging mistuned notes, we increased the difficulty level of the task by conducting a follow-up experiment (Experiment 2). In the Mistuned condition in Experiment 1, the incongruent melodies each contained a musical note that was not only mistuned by a quarter-tone, but was also a note that resulted in a tonal/syntactic violation. In Experiment 2, the tonal/syntactic violation was eliminated. New participants were tested.

## Experiment 2

### Methods

#### Participants

Twenty-six native English-speaking (12 females) and 22 native Cantonese-speaking (12 females) young adults were recruited from Northwestern University (16 English-speaking and 13 Cantonese-speaking) and the Nanyang Technological University of Singapore (6 English-speaking and 9 Cantonese-speaking). The Cantonese- and English-speaking groups did not differ in age [F(1, 46) = 2.0, p = 0.164, η*_p_* ^2^ = 0.042], education level [F(1, 46) = 0.512, p = 0.478, η*_p_* ^2^ = 0.011], or musical training level [F(1, 46) = 1.168, p = 0.285, η*_p_* ^2^ = 0.025]. Table 4 summarizes participants' demographic information.

**Table 4:** Demographic information and results for participants from Experiment 2.

	Native Cantonese-speaking *N* = 37	Native English-speaking *N* = 26
Off-beat Accuracy (SD)	86.93% (13.15)	89.05% (7.68)
Off-beat d′ (SD)	2.87 (1.18)	2.96 (.82)
Mistuned Accuracy (SD)	78.26% (8.41)	73.45% (13.08)
Mistuned d′ (SD)	1.82 (.59)	1.64 (.73)
Mean Age (SD)	20.64 (2.15)	21.59 (3.19)
Mean Year of Education (SD)	15.14 (1.86)	15.5 (2.09)
Mean Musical Training Level* (SD)	2.77 (1.77)	3.14 (1.08)

* See Table 1 for scale.

There was no significant group difference on any measure.

#### Methods and Materials

There were two conditions in this experiment, the Mistuned and Off-beat conditions. As in Experiment 1, there were congruent and incongruent melodies, with the 12 congruent melodies taken from Experiment 1. In the Mistuned condition, one note in each of the 12 melodies was mistuned by 50 cents (quarter-tone). The critical difference between this experiment and Experiment 1 is that the melodies in this condition contained no tonal/syntactic violation. By tonal/syntactic violation, we refer to notes that violate combinatorial rules that govern how notes should be integrated in a melody according to the Western tonal scale. In the Off-beat control condition, all notes in the melody were in-tune but one note contained a rhythmic incongruity, as in Experiment 1. In each condition, the participants performed a same-different AX discrimination task, indicating whether each pair of melodies was identical in every aspect. In each condition, there were 12 congruent and 12 incongruent melodies. The complete pairing of these two categories of melodies resulted in 48 trials for each condition, with 50% of same/different trials in each condition. Participants listened to each trial once in each condition. The order of presentation of the two conditions was counterbalanced across participants.

### Results and Discussion

For both conditions, we calculated accuracy and d′ scores [d′ = z(hit)−z(false alarm)] [46], and performed one-way ANOVA to compare performance across the two groups of participants. As in Experiment 1, no significant group difference was found in either the Mistuned [Accuracy: F(1, 46) = 2.208, p = 0.144, η*_p_* ^2^ = 0.046; d′: F(1, 46) = 0.846, p = 0.362, η*_p_* ^2^ = 0.018] or the Off-beat [Accuracy: F(1, 46) = 0.498, p = 0.484, η*_p_* ^2^ = 0.011; d′: F(1, 46) = 0.114, p = 0.737, η*_p_* ^2^ = 0.002] condition. Table 4 summarizes the results.

The similar pattern of results in this experiment and in the Mistuned condition in Experiment 1 suggests that Chinese and Western participants did not differ in their ability to attend to smaller pitch differences occurring in the context of a musical melody.

## Discussion

We found evidence of cultural differences in auditory processing. Specifically, speakers of a tone language show enhanced ability in processing musical pitch relative to those who speak a non-tone language. It is especially important to note that this difference remained after age, education, and musical training were controlled. Moreover, this difference is unlikely to be due to discarding attentional or motivational differences, because we found no difference in rhythmic perception (the Off-beat condition) between the two groups, even though both groups performed well above chance and far from ceiling in this condition. Our results are broadly consistent with recent studies showing cultural differences in visual perception [7] and other domains such as attentional control [47]. They are also consistent with studies suggesting the influence of musical training on lexical tone processing [32]. However, our results are the first to show that experience with lexical tones has an impact on musical pitch processing in listeners with no obvious musical pitch deficits, providing mirroring evidence for a double association of music and speech. We specifically examined musical pitch processing in musical melodies.

Throughout our study, we intentionally define musical pitch processing as the processing of musical melodies that requires the integration of local and longer distance pitch syntactic information. This definition acknowledges the fact that music in the real-world contains not only elements of isolated pitch patterns or pitch pairing relationships, but also phrasal structures governed by combinatorial rules. Languages that utilize intonational and lexical tones contain recognizable pitch patterns and pitch intervals (e.g., the word “today” in American English is spoken roughly in a minor third interval in citation form). Thus, it is challenging to restrict pitch interval tasks or brainstem encoding of frequency modulation to the domain of music, as they can also be applicable to language. In our view, it is relatively undisputable that melodic tasks that utilize musical syntactic rules from a particular tradition (e.g., Western tonal scale) should be attributed to the domain of music only (e.g., rules governing Western tonal music do not apply to English syntax). Thus, as we make claims about musical processing in the present study, we believe this definition is well justified.

It is worth noting that although the present study is the first to find significant results, previous studies have examined potential linguistic influence on musical pitch processing in musical melodies in Mandarin Chinese speakers but were unable to find a significant association [42], [23]. The discrepancies between our current significant results and the null findings in those studies could be due to the fact that Cantonese speakers were used in the current study. Unlike Mandarin, Cantonese contains six lexical tones with three levels tone that potentially impose greater demands on using contextual information for tone processing (see discussion below). All four pitch patterns in Mandarin have unique contour shapes. Furthermore, the current study has a much larger sample size, which might have afforded greater power for detecting significant differences.

The enhanced performance of Hong Kong participants becomes more evident when amusic participants from the two populations are considered. A major challenge of our study is to describe prevalence rates of amusia and to compare the performance of amusic individuals across cultures, due to the lack of an absolute standard, the lack of normality of distributions of the participant groups, and differences in relevant participant characteristics such as musical training across groups. We have adopted numerous approaches to overcome these difficulties, all of which pointed to superior musical pitch performance (and arguably lower prevalence of amusia) in the Hong Kong group. When focused on a criterion-based approach using three arbitrary cutoff values, we found a higher percentage of Canadian participants to perform below the cutoffs (Table 2). When focused on the lowest 5% of each of the population (a commonly-found prevalence rate of amusia in Western populations), we found Hong Kong participants to significantly outperform the Canadian participants even after differences in musical training were adjusted statistically. In other words, regardless of whether the general population or the amusic group was the focus, speakers of a tone language showed enhanced musical pitch abilities relative to their non-tone language counterparts. These enhanced musical pitch abilities may be related to why fewer Hong Kong amusics reported to have difficulty with vocal music in general (Table 3).

The critical question to consider here is why tone language speakers possess better musical pitch abilities. As our results suggest, the better musical abilities we observed are highly specific. That is, it is not a general musical advantage that also includes rhythm. It is also not a general pitch advantage that includes differentiating smaller pitch differences. Rather, it is specific to integrating pitch information across a melody. Although previous studies have argued for a higher prevalence rate of absolute pitch in tone language speakers [48], absolute pitch plays less of a role in our results as evaluations of the musical phrase (and context) are essential to the participants' success. An explanation of our findings would then require a focus on how musical context is being utilized. We postulate that our results could be explained by a mechanism we call “Perceptual Normalization of Tonal Categories.” Under this proposal, tonal categories include musical tones (e.g., a tonic in an A-major scale), lexical tones (e.g., a high level tone in Mandarin), or an intonational tone (e.g., H tone in a phrase) [49]. The perceptual normalization aspect of our proposal proffers that to arrive at tonal categories (musical, lexical, or intonational), listeners need contextual information. For example, to determine that the fourth note/syllable in a five-note melodic/phrasal sequence is a tonic or level tone, listeners need to attend to notes 1, 2, 3, and 5 in the sequence. That is, listeners' perception of the 4^th^ note as a tonic or a mid-level tone is determined by its relationship with the surrounding notes. Previously, we found evidence of such a perceptual normalization process in lexical tones, which we argued is a mechanism for compensating for talker variation [50]. The important claim of this proposal is that although there are domain-specific representations regarding music and speech perception, as evidenced by double dissociations found in acquired amusic and aphasic patients [51], the process of perceptual normalization is domain-general. If this is the case, exposure to one type of tonal system (musical tone, lexical tone, or intonational tones) should facilitate the normalization of other types of tones. Our proposal here overlaps with the “Shared Syntactic Resource Integration Hypothesis” [52] in arguing for shared processing resources for music and language while maintaining that representations for specific music and language systems are separate. Specific to our Out-of-Key condition, all participants had knowledge (representations) of Western musical tones and intonational tones through acquisition/exposure of their specific musical and linguistic systems, but only the Hong Kong subjects had additional knowledge of a lexical tone system. Under our proposal, this additional experience gave them an advantage in judging (processing) the fit of the musical tones in our stimuli with the Western tonal key system.

Our proposal speaks specifically to listeners' ability to integrate local and long-distance pitch information across a melody, but it does not necessarily predict better ability to encode small musical pitch differences, as some recent behavioral and neurophysiological data also predict [37], [23], [34], [35], [39]. The two groups of participants performed similarly in conditions in which they were required to process small musical pitch differences (within one semitone) in a larger musical context in the Mistuned condition in Experiment 1 and Experiment 2. These results are consistent with prior studies that did not find Mandarin-speaking participants to show better fine-grained pitch perception abilities [41], [23].

We are also intrigued by the occurrence of amusia in some of our Hong Kong participants. If our proposal of perceptual normalization of tonal categories is correct, difficulty in the normalization of musical tones should also affect the normalization of lexical tones, which may ultimately lead to linguistic processing difficulties at the word, sentence, and even discourse levels in tone language speakers. In fact, Nan, Sun, and Peretz (2010) [42] and Jiang et al. (2010) [43] respectively found impaired perception of lexical tones and intonation contour in Mandarin amusic listeners. These findings join other studies implicating the importance of cultural contextualization of the neural bases of higher-level functions and behavioral disorders, such as dyslexia [53], [54] and reading [23], [53] and psychiatric disorders [55].

Several limitations of the present study are worth mentioning. First, a recent study by Dediu and Ladd [56] demonstrates a link between the population frequency of two genes (the derived alleles of *ASPM* and *MCPH1*) and the incidence of lexical tone in the languages spoken by different populations. Thus, tone language speakers may possess an advantage in processing pitch that is irrespective of their experiences with speaking a tone language. Regardless of whether genes and/or experience are explanatory factors, the finding remains that cultural differences are associated with pitch processing. If genetic factors explain our results, whether an advantage in tonal processing of one system is gained from exposure to another system would remain to be investigated. Second, even though the level of musical training was controlled for statistically, Hong Kong participants might have received a qualitatively different type of musical training that gave them an advantage, although it is difficult to explain why such qualitatively different training would only affect pitch but not rhythm perception. Third, there might be differences in everyday music exposure and usage by the two groups. Specifically, although Western tonal music is very prevalent in the Hong Kong environment and in musical education, music written in the Chinese pentatonic system still plays a role in the culture. However, we have no explanation as to why exposure to pentatonic music might have provided an advantage to the Hong Kong subjects, if this is in fact a factor. Fourth, there were numerous other factors we were unable to control, including more relevant factors such as internet usage, testing environment, listening equipment, and seemingly less relevant factors such as height, weight, diet, climate, and other subject-internal, environmental, and cultural factors. We have no reason to believe that internet speed and available everyday audio equipment differed between Hong Kong and Montreal, as both are highly developed cities. We also have no reason to believe that the two groups, being composed of predominately younger university students in top world universities, differed systematically in IQ, cognitive abilities, or other measurable characteristics that could explain the results. It is worth pointing out that although cultural differences of working memory between Cantonese- and English-speaking adults were found [57], they were attributed to the structural characteristics (e.g., syllable length and consonant-vowel structures) of the language to be remembered (e.g., Cantonese digits are mono-syllabic and have simpler syllable structures) [58].

While philosophers and psychologists have long debated the extent to which language exerts influence on perception [4], [59], experimental evidence has only recently been a medium for exploration [60], [61]. We have found a potential influence of linguistic experiences on the perception of musical melodies in a large population of listeners. Thus, our findings here represent a starting point for a series of studies that will provide a more comprehensive picture of cultural differences in basic and higher-level auditory processing. In particular, future studies should include investigations of neuroanatomical differences between tone and non-tone language speakers with respect to musical and non-lexical pitch processing, investigations of the developmental time course of such differences, as well as genetic and environment interactions, perhaps with a focus on *ASPM* and *MCPH1*.

## Supporting Information

Figure S1 Distribution characteristics of Hong Kong participants' performance on the Online Amusia test. Panel (a) shows Global Scores; panels (b) to (d) show Off-beat, Mistuned, and Out-of-Key results respectively, divided by age groups (40 years).
(TIF)

Table S1 Hong Kong Participant Characteristics.
(DOC)

Table S2 Performance of Hong Kong Participants on the Online Amusia Test.
(DOC)

Supporting Information S1 Normative Data of the On-Line Identification Test of Congenital Amusia (Hong Kong Version).
(DOC)

## References

[1] Williams R (1976). Keywords: A vocabulary of culture and society.

[2] Nisbett RE, Masuda T (2003). Culture and point of view.. Proc Natl Acad Sci U S A.

[3] Han S, Northoff G (2008). Culture-sensitive neural substrates of human cognition: a transcultural neuroimaging approach.. Nat Rev Neurosci.

[4] Whorf BL, Carroll JB (1956). Language, thought, and reality: Selected writings of Benjamin Lee Whorf.

[5] Masuda T, Nisbett RE (2001). Attending holistically versus analytically: comparing the context sensitivity of Japanese and Americans.. J Personal Soc Psychol.

[6] Chan AHD, Tan LH, Kay P, Khong PL, Yip LK (2008). Language affects patterns of brain activation associated with perceptual decision.. Proc Natl Acad Sci U S A.

[7] Chua HF, Boland JE, Nisbett RE (2005). Cultural variation in eye movements during scene perception.. Proc Natl Acad Sci U S A.

[8] Triandis HC (1995). Individualism and collectivism.

[9] Markus H, Kitayama S (1991). Culture and the self: implications for cognition, emotion, and motivation.. Psychol Rev.

[10] Singelis TM (1994). The measurement of independent and interdependent self-construals.. Pers Soc Psychol Bull.

[11] Nisbett RE, Peng K, Choi I, Norenzayan A (2001). Culture and systems of thought: holistic versus analytic cognition.. Psychol Rev.

[12] Yip MJ (2002). Tone.

[13] Peretz I, Gosselin N, Tillmann B, Cuddy LL, Gagnon B (2008). On-line identification of congenital amusia.. Music Percept.

[14] Fry DB (1948). An experimental study of tone deafness.. Speech.

[15] Kalmus H, Fry DB (1980). On tune deafness (dysmelodia): frequency, development, genetics and musical background.. Ann Hum Genet.

[16] Trehub SE, Hannon EE (2006). Infant music perception: domain-general for domain-specific mechanisms?. Cognition.

[17] Hyde KL, Zatorre RJ, Griffiths TD, Lerch JP, Peretz I (2006). Morphometry of the amusic brain: a two-site study.. Brain.

[18] Hyde KL, Lerch JP, Zatorre RJ, Griffiths TD, Evans AC (2007). Cortical thickness in congenital amusia: When less is better than more.. J Neurosci.

[19] Mandell J, Schulze K, Schlaug G (2007). Congenital amusia: an auditory-motor feedback disorder?. Restor Neurol Neurosci.

[20] Peretz I, Cummings S, Dubé M-P (2007). The genetics of congenital amusia (tone deafness): A family-aggregation study.. Am J Hum Genet.

[21] Drayna D, Manichaikul A, de Lange M, Snieder H, Spector T (2001). Genetic Correlates of Musical Pitch Recognition in Humans.. Science.

[22] Brugge JF, Popper AN, Fay RR (1992). An overview of central auditory processing.. The mammalian auditory pathway: Neurophysiology.

[23] Peretz I, Nguyen S, Cummings S (2011). Tone language fluency impairs pitch discrimination. Front.. Pyschology.

[24] Milovanov R, Tervaniemi M (2011). The Interplay between Musical and Linguistic Aptitudes: A Review. Front.. Psychology.

[25] Schön D, François C (2011). Musical Expertise and Statistical Learning of Musical and Linguistic Structures. Front.. Psychology.

[26] Ettlinger M, Margulis EH, Wong PCM (2011). Implicit Memory in Music and Language. Front.. Psychology.

[27] Tillmann B, Burnham D, Nguyen S, Grimault N, Gosselin N (2011). Congenital amusia (or tone-deafness) interferes with pitch processing in tone languages. Front.. Psychology.

[28] Hoch L, Poulin-Charronnat B, Tillmann B (2011). The Influence of Task-Irrelevant Music on Language Processing: Syntactic and Semantic Structures. Front.. Psychology.

[29] Patel AD (2011). Why would musical training benefit the neural encoding of speech? The OPERA hypothesis. Front.. Psychology.

[30] Lee C-Y, Hung T-H (2008). Identification of Mandarin tones by English-speaking musicians and nonmusicians.. J Acoust Soc Am.

[31] Wong PCM, Perrachione TK (2007). Learning pitch patterns in lexical identification by native English-speaking adults.. Appl Pscyholinguist.

[32] Wong PCM, Skoe E, Russo NM, Dees T, Kraus N (2007). Musical experience shapes human brainstem encoding of linguistic pitch patterns.. Nat Neurosci.

[33] Wong PCM, Warrier CM, Penhune VB, Roy AK, Sadehh A (2008). Volume of left Heschl's Gyrus and linguistic pitch learning.. Cereb Cortex.

[34] Bidelman GM, Gandour JT, Krishnan A (2011a). Cross-domain effects of music and language experience on the representation of pitch in the human auditory brainstem.. J Cogn Neurosci.

[35] Bidelman GM, Gandour JT, Krishnan A (2011b). Musicians and tone-language speakers share enhanced brainstem encoding but not perceptual benefits for musical pitch.. Brain Cogn.

[36] Jeng FC, Hu J, Dickman B, Montgomery-Reagan K, Tong M (2011). Cross-linguistic comparison of frequency-following responses to voice pitch in American and Chinese neonates and adults.. Ear Hear.

[37] Pfordresher PQ, Brown S (2009). Enhanced production and perception of musical pitch in tone language speakers.. Atten Percept Psychophys.

[38] Hove MJ, Sutherland ME, Krumhansl CL (2010). Ethnicity effects in relative pitch.. Psychon Bull Rev.

[39] Giuliano RJ, Pfordresher PQ, Stanley EM, Narayana S, Wicha NY (2011). Native experience with a tone language enhances pitch discrimination and the timing of neural responses to pitch change. Front.. Psychology.

[40] Lee CY, Lee YF (2010). Perception of musical pitch and lexical tones by Mandarin-speaking musicians.. J Acoust Soc Am.

[41] Bent T, Bradlow AR, Wright BA (2006). The influence of linguistic experience on the cognitive processing of pitch in speech and nonspeech sounds.. J Exp Psychol Hum Percept Perform.

[42] Nan Y, Sun Y, Peretz I (2010). Congenital amusia in speakers of a tone language: association with lexical tone agnosia.. Brain.

[43] Jiang C, Hamm JP, Lim VK, Kirk IJ, Yang Y (2010). Processing melodic contour and speech intonation in congenital amusics with Mandarin Chinese.. Neuropsychologia.

[44] Peretz I, Champod S, Hyde K (2003). Varieties of musical disorders: the Montreal battery of evaluation of amusia.. Ann NY Acad Sci.

[45] Hays W (1994). Statistics.

[46] Green DM, Swets JA (1966). Signal detection theory and psychophysics.

[47] Hedden T, Ketay S, Aron A, Markus HR, Gabrieli JDE (2008). Cultural influences on neural substrates of attentional control.. Psychol Sci.

[48] Deutsch D, Henthorn T, Marvin E, Xu H (2006). Absolute pitch among American and Chinese conservatory students: prevalence differences, and evidence for a speech-related critical period.. J Acoust Soc Am.

[49] Pierrehumbert J (1979). The perception of fundamental frequency declination.. J Acoust Soc Am.

[50] Wong PCM, Diehl RL (2003). Perceptual normalization of inter- and intra-talker variation in Cantonese level tones.. J Speech Lang Hear Res.

[51] Peretz I (2006). The nature of music from a biological perspective.. Cognition.

[52] Patel AD (2003). Language, music, syntax and the brain.. Nat Neurosci.

[53] Siok WT, Niu ZD, Jin Z, Perfetti CA, Tan LH (2008). A structural-functional basis for dyslexia in the cortex of Chinese readers.. Proc Natl Acad Sci U S A.

[54] Siok WT, Perfetti CA, Jin Z, Tan LH (2004). Biological abnormality of impaired reading is constrained by culture.. Nature.

[55] Kleinman A (1981). Patients and healers in the context of culture.

[56] Dediu D, Ladd DR (2007). Linguistic tone is related to the population frequency of the adaptive haplogroups of two brain size genes, ASPM and Microcephalin.. Proc Natl Acad Sci U S A.

[57] Stigler JW, Lee SY, Stevenson HW (1986). Digit memory in Chinese and English: Evidence for a temporally limited store.. Cognition.

[58] Cheung H, Kemper S, Leung E (2000). A Phonological Account for the Cross-Language Variation in Working Memory Processing.. The Psychological Record.

[59] Kay P, Kempton W (1984). What is the Sapir-Whorf Hypothesis?. Am Anthropol.

[60] Tan LH, Chan AHD, Kay P, Khong PK, Yip L (2008). Language affects patterns of brain activation associated with perception.. Proc Natl Acad Sci USA.

[61] Winawer J, Witthoft N, Frank MC, Wu L, Wade A (2007). The Russian blues: effects of language on color discrimination.. Proc Natl Acad Sci USA.

