# Supplementary figures and images for: Effects of Culture on Musical Pitch Perception
Source: PLoS One. 2012 Apr 11;7(4):e33424. doi: 10.1371/journal.pone.0033424 (PMC3324485; doi:10.1371/journal.pone.0033424)

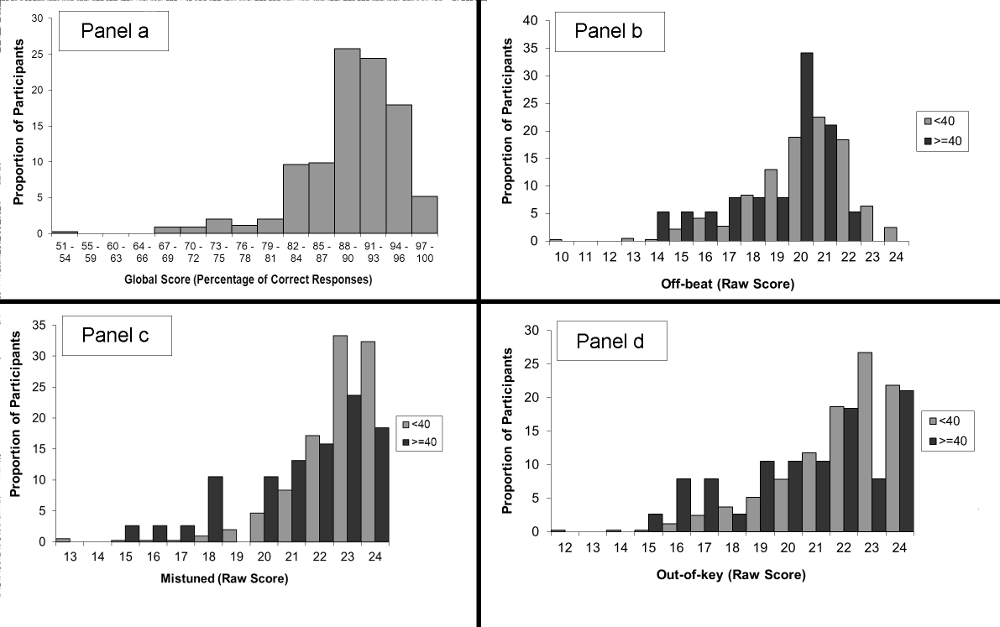

Supplement: Figure S1 — Distribution characteristics of Hong Kong participants' performance on the Online Amusia test. Panel (a) shows Global Scores; panels (b) to (d) show Off-beat, Mistuned, and Out-of-Key results respectively, divided by age groups (40 years). (TIF) [file pone.0033424.s001.tif]
