# Supplementary material for: Effects of Culture on Musical Pitch Perception
Source: PLoS One. 2012 Apr 11;7(4):e33424. doi: 10.1371/journal.pone.0033424 (PMC3324485; doi:10.1371/journal.pone.0033424)
Supplement: Table S1 — Hong Kong Participant Characteristics. (DOC) [file pone.0033424.s002.doc]

**Table S1. Hong Kong Participants Characteristics.**

|  | **Older** | | | **Younger** | | | **All** |
| --- | --- | --- | --- | --- | --- | --- | --- |
|  | Non-Amusic *N*=37 | Amusic *N*=1 | All Old  *N*=38 | Non-Amusic  *N*=392 | Amusic *N*=16 | All Young  *N*=408 | *N*=446 |
| Male/female (% female) | 14/23 (62.2) | 0/1 | 14/24 (63.2) | 135/257 (65.6) | 6/8 (57.1) | 141/267 (65.4) | 154/291 (65.2) |
| Mean age (range) | 46.8 (40-71) | 44 | 46.8 (40-71) | 23.5 (18-39) | 24.9 (20-34) | 23.5 (18-39) | 25.5 (18-71) |
| Mean education year (range) | 20.8 (10-43) | 37 | 21.3 (10-43) | 16.0 (7-33) | 15.9 (9-24) | 16.0 (7-33) | 16.4 (7-43) |
| Mean Musical training level | 3.3 (1-5) | 4 (1-5) | 3.3 (1-5) | 4.1 (1-5) | 3.7 (1-5) | 4.1 (1-5) | 4.0 (1-5) |
| Unmusical by self-report | 8.1% | 100.0% | 10.5% | 13.8% | 62.5% | 15.7% | 15.2% |
| Dyslexia | 2.8% (1/36) | 0% | 2.7% (1/37) | 0.8% (3/389) | 0% | 0.7% (3/405) | 0.9% (4/442) |
| Speech disorders | 5.6% (2/36) | 0% | 5.4% (2/37) | 2.1% (8/389) | 0% | 2.0% (8/405) | 2.3% (10/442) |
| Spatial orientation problems | 8.3% (3/36) | 0% | 8.1% (3/37) | 2.6% (10/389) | 0% | 2.5% (10/405) | 2.9% (13/442) |
| Problems in maths | 5.6% (2/36) | 0% | 5.4% (2/37) | 3.3% (13/389) | 6.3% (1/16) | 3.5% (14/405) | 3.6% (16/442) |
| Attentional problems | 5.6% (2/36) | 0% | 5.4% (2/37) | 3.3% (13/389) | 0% | 3.2% (13/405) | 3.4% (15/442) |
| Memory problems | 8.3% (3/36) | 0% | 8.1% (3/37) | 3.6% (14/389) | 0% | 3.5% (14/405) | 3.8% (17/442) |
| Unable to detect when someone sings out-of-tune | 2.7% (1/37) | 100% (1/1) | 5.3% (2/38) | 4.4% (17/388) | 25.0% (4/16) | 5.2% (21/404) | 5.2% (23/442) |
| Can rarely recognize a very familiar melody without the help of lyrics | 2.7% (1/37) | 100% (1/1) | 5.3% (2/38) | 2.3% (9/390) | 12.5% (2/16) | 2.7% (11/406) | 2.9% (13/444) |
| Sings out-of-tune | 18.9% (7/37) | 100% (1/1) | 21.1% (8/37) | 29.5% (114/387) | 75.0% (12/16) | 31.3% (126/403) | 30.5% (134/440) |

Older participants were at least 40 years old. Younger participants were between 18 and 40 years old. Behavioral/Cognitive deficits (e.g., dyslexia) are by self-report only.
