# Supplementary material for: Effects of Culture on Musical Pitch Perception
Source: PLoS One. 2012 Apr 11;7(4):e33424. doi: 10.1371/journal.pone.0033424 (PMC3324485; doi:10.1371/journal.pone.0033424)
Supplement: Table S2 — Performance of Hong Kong Participants on the Online Amusia Test. (DOC) [file pone.0033424.s003.doc]

**Table S2.** Performance of Hong Kong Participants on the Online Amusia Test.

|  |  | **Older** |  |  | **Younger** |  | **All** |
| --- | --- | --- | --- | --- | --- | --- | --- |
|  | Non-Amusic *N*=37 | Amusic *N*=1 | All Old  *N*=38 | Non-Amusic  *N*=392 | Amusic *N*=16 | All Young  *N*=408 | *N*=446 |
| Off-beat (SD) | 80.3% (8.8) | 62.5% | 79.4% (9.1) | 84.6% (8.1) | 69.0% (11.1) | 84.0% (8.8) | 83.6% (8.7) |
| Mistuned (SD) | 89.5% (10.2) | 83.3% | 89.0% (10.1) | 95.0% (5.1) | 76.6% (12.2) | 94.2% (6.6) | 93.8% (7.1) |
| Out-of-key (SD) | 86.9% (10.8) | 62.5% | 86.3% (11.4) | 91.9% (7.4) | 71.1% (10.0) | 91.1% (8.5) | 90.7% (8.9) |
| Global score (SD) | 85.5% (6.8) | 69.4% | 84.9% (7.2) | 90.5% (4.4) | 72.2% (6.6) | 89.8% (5.7) | 89.4% (6.0) |
| 2SD Cut-off |  | 70.5% |  |  | 78.4% |  | 77.4% |
| % Amusic/  Prevalence |  | 2.6% (1/38) |  |  | 3.9% (16/408) |  | 4.7% (21/446) |

The 2 standard deviations (SD) cutoff for amusia is based on the Global Score.
