# Supplementary material for: Effects of Culture on Musical Pitch Perception
Source: PLoS One. 2012 Apr 11;7(4):e33424. doi: 10.1371/journal.pone.0033424 (PMC3324485; doi:10.1371/journal.pone.0033424)
Supplement: Supporting Information S1 — Normative Data of the On-Line Identification Test of Congenital Amusia (Hong Kong Version). (DOC) [file pone.0033424.s004.doc]

**SUPPORTING INFORMATION S1: Normative Data of the On-Line Identification Test of Congenital Amusia (Hong Kong Version)**

Because the Hong Kong version of the On-Line Identification of Congenital Amusia test (henceforth the “HK Online Test”) was not previously normed, we report here the psychometric data of this version including all participants (all who were 18 years or above). We then report more specifically additional comparisons between Hong Kong and Canadian participants who were younger than 40 years old. As discussed in the main document, after the online test, participants were asked to answer a series of questions concerning their health, musical, and educational history, and relevant responses are reported here. Note that not all participants answered all questions, which resulted in missing data points and differences in degrees of freedom reported below.

*Results: Normative Data of the Hong Kong version of the On-Line Identification of Congenital Amusia Test*

A statistical comparison between results from the Hong Kong and Canadian [13] participants were discussed in the main document, and additional information will be discussed in the next section. Here, we report results from all Hong Kong participants (Table S1 and Figure S1).

*Distribution Characteristics of the Online Test.* Figure S1 (panel a) shows the distribution of the Global Scores (all participants were combined), which was non-normal [Kolmogorov-Smirnov Z = 3.075, p < .001]. Similar to Peretz et al. (2008) [13], the distribution is negatively skewed and is sensitive for detecting participants with low performance. Note that although neither the current, nor the Peretz et al. (2008) distributions were normal, the use of parametric tests are justified due to our large sample sizes [45]. Only one out of the 446 participants scored perfectly. Panels b, c, and d of Figure S1 respectively show the distribution of scores for the Off-beat, Mistuned, and Out-of-Key conditions, divided by age group.

*Definition of Amusia and Prevalence.* Following Peretz et al. (2008) [13], we define amusia based on the criterion of two standard deviations below the mean. Table S2 shows the cutoff scores and prevalence. For younger participants, the cutoff was at 78.4%; 3.9% of the younger participants scored below this cutoff. This prevalence rate is lower than the Canadian population tested previously (5.2% for participants between 18 and 40 years old), even though the cutoff score is higher for the Hong Kong participants. For the older participants, the cutoff score was substantially lower (70.5%). Note that relatively fewer of the older participants completed the test so the low prevalence rate (2.6%) should be interpreted cautiously. If all participants regardless of age were considered, the 2 SD cutoff would be 77.4% with a prevalence rate of 4.7%. As discussed in the main document, the use of the 2 SD cutoff criterion is simply conventional, and we are aware that a unit of SD does not translate into a fixed percentage of population when the distribution is not normal. Also discussed in the main document is the fact that Hong Kong amusic individuals outperformed their Canadian counterparts.

*Gender Differences.* Similar to Peretz et al. (2008) [13], we found no gender difference in performance on the Hong Kong Online Test (Global Score) by our participants from Hong Kong [F (1,444) = .062, p = .804)].

*Musical Training.* Similar to Peretz et al. (2008) [13], musical training positively correlated with all measures of the Hong Kong Online Test [Off-beat: r (436) = .120, p = .012; Mistuned: r (436) = .143, p = .003; Out-of-Key: r (436) = .257, p < .001; Global: r (436) = .243, p < .001), as well as negatively correlated with age (r (436) = -.230, p < .001).

*Age and Education.* Age was negatively correlated with some measures of the Hong Kong Online Test [Off-beat: r (444) = -.191, p < .001; Mistuned: r (444) = -.189, p < .001; Out-of-Key: r (444) = -.162, p < .001; Global: r (444) = -.248, p < .001]. Younger participants tended to perform better.

Because age and education were positively correlated [r (442) = .488, p < .001] in our sample, years of education was also significantly negatively correlated with some measures of the online test (Off-beat: r (442) = -.064, p = .181; Mistuned: r (442) = -.220, p < .001; Out-of-Key: r (442) = -.135, p = .004; Global: r (442) = -.185, p < .001).

*Cohort (Age) Effects.* Peretz et al. (2008) [13] found systematic differences in how participants of different ages performed. Following their study, we divided our subjects into two groups using 40 years of age as a cutoff. A 2 x 3 (age group x condition) revealed a main effect of condition [F (2, 888) = 72.414, p < 001) and age group [F (1, 444) = 24.139, p < 001], but no significant interaction [F (2, 888) = .066, p = .936]. Younger participants consistently outperformed older participants across all conditions.

Although older participants tend to be more educated [F (1,442) = 60.752, p < .001], they had less musical training [F (1, 436) = 15.243, p < .000], which corroborated the correlation results reported above and is consistent with findings by Peretz and colleagues [44].

*Results: Younger Participants Only*

This section provides additional information regarding comparisons between the Hong Kong and the Canadian participants that were not reported in the main document. Because very few Hong Kong participants older than 40 years old participated, only younger participants are included. We also provide additional correlational analyses with the Hong Kong and Canadian younger participants combined.

Table 1 in the main document shows basic characteristics of these two groups of younger participants. Although younger participants were chosen, the Hong Kong group was still younger than the Canadian group by about one year [F (1, 560) = 5.699, p = .017]. Although the Canadian group was in school for about one year longer [F (1, 560) = 11.717, p = .001], they had less musical training than the Hong Kong participants by about one year [F (1, 560) = 75.136, p < .001].

We also performed a series of correlational analyses (p values were not corrected) using Pearson r to explore the relationships among age, education level, musical training, and performance on the online test; only significant results are reported here. The analyses here included those when the two groups of younger participants were combined. Age was significantly correlated with education [Pearson r (559) = .546, p < .001] but negatively correlated with musical training [r (544) = -.200, p < .001], in all test conditions [Off-beat: r (560) = -.137, p = .001; Mistuned: r (560) = -.124, p = .003; Out-of-Key: r (560) = -.102, p = .016; Global: r (560) = -.163, p < .001]. Most importantly, musical training was positively correlated with all measures (Off-beat: r (544) = .106, p = .013; Mistuned: r (544) = .162, p < .001; Out-of-Key: r (544) = .322, p < .001; Global: r (544) = .282, p < .001]. Education was not correlated with any measures of the amusia test. Because of these correlational characteristics, and because the two groups of participants differed in some basic characteristics, we reported in the main document results from an ANCOVA that controlled for age, education, and musical training.

**SUPPORTING REFERENCES**

45. Hays W (1994) Statistics. Forth Worth, TX: Harcourt.
